# Development and validation of prognostic nomogram for germ cell testicular cancer patients

**DOI:** 10.18632/aging.104063

**Published:** 2020-11-02

**Authors:** Weipu Mao, Jianping Wu, Qingfang Kong, Jian Li, Bin Xu, Ming Chen

**Affiliations:** 1 Department of Urology, People’s Hospital of Putuo, Shanghai 200060, China; 2 Department of Urology, Affiliated Zhongda Hospital of Southeast University, Nanjing 210009, China; 3 Department of Nosocomial Infection, Affiliated Zhongda Hospital of Southeast University, Nanjing 210009, China; 4 Department of Urology, The People’s Hospital of Jinhu, Huaian 211600, Jiangsu Province, China

**Keywords:** germ cell testicular cancer, prognostic nomogram, overall survival, cancer-specific survival, SEER

## Abstract

The purpose of our study was to establish a reliable and practical nomogram based on significant clinical factors to predict the overall survival (OS) and cancer-specific survival (CSS) of patients with germ cell testicular cancer (GCTC). Patients diagnosed with GCTC between 2004 and 2015 were obtained from the SEER database. Nomograms were constructed using the R software to predict the OS and CSS probabilities and the constructed nomograms were validated and calibrated. A total of 22,165 GCTC patients were enrolled in the study, including the training cohort (15,515 patients) and the validation cohort (6,650 patients). In the training cohort, multivariate Cox regression showed that age, race, AJCC stage, SEER stage and surgery were independent prognostic factors for OS, while age, race, AJCC stage, TM stage, SEER stage and radiotherapy were independent prognostic factors for CSS. Based on the above Cox regression results, we constructed prognostic nomograms of OS and CSS in GCTC patients and found that the OS nomograms had higher C-index and AUC compared to TNM stage in the training and validation cohorts. In addition, in the training and external validation cohorts, the calibration curves showed a good consistency between the predicted and actual 3-, 5- and 10-year OS and CSS rates of the nomogram. The current prognostic nomogram can provide a personalized risk assessment for the survival of GCTC patients.

## INTRODUCTION

Testicular cancer (TC) is a rare malignant tumor in the genitourinary system, accounting for about 5% of genitourinary tumors [1]. In 2018, 71,105 new cases of TC (1.7% of male incidence) and 9,507 deaths (0.2% of male mortality) were diagnosed worldwide [2]. Despite the lower overall incidence, TC is the most common malignant tumor in men aged 15-34 years [3].

Germ cell TC (GCTC) is the most common type of TC. GCTC mostly occurs on one side and only about 1% on both sides [4]. The main risk factor for GCTC is cryptorchidism, which occurs in 2-5% of boys born at term [5]. The other risk factors include gonadal dysgenesis and genetic diseases such as Down’s syndrome [6, 7]. Although the overall 10-year cancer-specific mortality (CSM)-free survival rate in patients with GCTC is approaching 95%, the incidence has increased significantly over the past 30 years [8, 9]. Therefore, further research is important to determine the predictors that may affect the long-term survival of GCTC patients.

The American Joint Committee on Cancer (AJCC) tumor node metastasis (TNM) staging system is widely used to evaluate the prognosis of patients with GCTC. However, some other factors such as age, race, SEER stage, surgery and radiotherapy can also affect the outcome of GCTC patients. The nomogram based on the equations derived from the regression coefficients of each variable integrates many prognostic factors, which can more accurately predict the individual survival [10]. The nomogram can incorporate important clinicopathological and demographic variables in clinical practice to create a more comprehensive prognostic evaluation system.

In this study, we analyzed the clinicopathological features and prognostic factors of GCTC patients using the Surveillance, Epidemiology, and End Results (SEER) database. Based on the results of survival analysis, we further developed and validated the prognostic nomogram for patients with GCTC to better predict the patient's prognosis.

## RESULTS

### Demographic and clinicopathologic characteristics

From 2004 to 2015, our study cohort included 22,165 eligible GCTC patients, including 15,515 patients in the training cohort and 6,650 patients in the validation cohort. Table 1 shows the demographic and clinical characteristics of patients with GCTC. In the entire cohort, the majority of GCTC patients were white (90.3%), and the age of onset was concentrated between 21-40 years (65.8%). The most common types were AJCC I stage (77.2%), T1 stage (66.9%), N0 stage (80.3%), M0 stage (90.8%) and localized stage (72.2%). In addition, nearly 99.9% of patients received surgery while only 20.2% received radiotherapy.

**Table 1:** Baseline demographic and clinical characteristics with testicular germ cell tumor (TGCT) patients in our study.

**Characteristic**	**Total No. (%)**	**The training cohort**		**The validation cohort**
		**No. (%)**		**No. (%)**
Total	22165	15515 (70.0)		6650 (30.0)
Age at diagnosis				
0-20	1809 (8.2)	1238 (8.0)		571 (8.6)
21-40	14569 (65.7)	10211 (65.8)		4358 (65.5)
41-60	5334 (24.1)	3739 (24.1)		1595 (24.0)
> 60	453 (2.0)	327 (2.1)		126 (1.9)
Race				
White	20026 (90.3)	14029 (90.4)		5997 (90.2)
Black	586 (2.6)	402 (2.6)		184 (2.8)
Others	1553 (7.0)	1084 (7.0)		469 (7.1)
AJCC stage				
I	17121 (77.2)	11997 (77.3)		5124 (77.1)
II	2519 (11.4)	1753 (11.3)		766 (11.5)
III	2525 (11.4)	1765 (11.4)		760 (11.4)
T stage				
T1	14829 (66.9)	10367 (66.8)		4462 (67.1)
T2	6132 (27.7)	4302 (27.7)		1830 (27.5)
T3	1071 (4.8)	761 (4.9)		310 (4.7)
T4	133 (0.6)	85 (0.5)		48 (0.7)
N stage				
N0	17804 (80.3)	12457 (80.3)		5347 (80.4)
N1	2145 (9.7)	1482 (9.6)		663 (10.0)
N2	1250 (5.6)	898 (5.8)		352 (5.3)
N3	966 (4.4)	678 (4.4)		288 (4.3)
M stage				
M0	20121 (90.8)	14087 (90.8)		6034 (90.7)
M1	2044 (9.2)	1428 (9.2)		616 (9.3)
SEER stage				
Localized	16001 (72.2)	11193 (72.1)		4808 (72.3)
Regional	4085 (18.4)	2881 (18.6)		1204 (18.1)
Distant	2079 (9.4)	1441 (9.3)		638 (9.6)
Surgery				
No	19 (0.1)	13 (0.1)		6 (0.1)
Yes	22146 (99.9)	15502 (99.9)		6644 (99.9)
Radiotherapy				
Yes	4472 (20.2)	3155 (20.3)		1317 (19.8)
No	17693 (79.8)	12360 (79.7)		5333 (80.2)

Abbreviations: AJCC, American Joint Committee on Cancer; SEER, Surveillance, Epidemiology, and End Results.

Percentages may not total 100 because of rounding.

### Survival of patients with GCTC

By analyzing the Kaplan-Meier curve with a log-rank test we found that age at diagnosis, race, AJCC stage, T stage, N stage, M stage, SEER stage, surgery and radiotherapy (All p<0.05) were associated with OS and CSS of GCTC patients (Table 2). In the whole cohort, the 3 -, 5- and 10-year OS of GCTC patients were 96.4%, 95.6% and 93.6%, respectively, and the 3 -, 5- and 10-year CSS were 97.7%, 97.4% and 97.2%, respectively. We found that patients aged 21-40 and received chemotherapy had a higher survival rate.

**Table 2:** Kaplan–Meier analysis overall survival (OS) and cancer-specific survival (CSS) for testicular germ cell tumor (TGCT) patients.

**Characteristic**	**Overall Survival (OS)**					**Cancer-Specific Survival (CSS)**				
	**3-year OS %**	**5-year OS %**	**10-year OS %**	**Kaplan-Meier**		**3-year CSS %**	**5-year CSS %**	**10-year OS %**	**Kaplan-Meier**	
				**Log Rank χ2 test**	**P value**				**Log Rank χ2 test**	**P value**
All	96.4	95.6	93.6			97.7	97.4	97.2		
Age at diagnosis				260.021	<.001				29.805	<.001
0-20	95.9	95.6	94.2			97.1	96.7	96.5		
21-40	97.0	96.2	95.0			97.9	97.6	97.4		
41-60	95.7	94.9	91.7			97.6	97.4	97.0		
> 60	89.3	83.3	67.4			95.1	93.5	93.0		
Race				17.099	<.001				9.473	.009
White	96.5	95.7	93.7			97.8	97.5	97.2		
Black	93.1	92.6	89.4			96.4	95.9	95.1		
Others	96.6	95.6	94.5			97.6	97.1	96.9		
AJCC stage				1594.930	<.001				1865.778	<.001
I	98.6	97.9	96.1			99.4	99.2	99.1		
II	97.4	96.4	94.9			98.6	98.3	98.1		
III	81.3	79.9	76.3			85.6	84.8	83.8		
T stage				410.212	<.001				427.797	<.001
T1	97.5	96.6	94.8			98.4	98.2	97.9		
T2	96.2	95.7	93.2			97.5	97.3	97.1		
T3	89.5	87.2	85.3			93.0	91.9	91.5		
T4	71.0	68.7	68.7			76.8	75.5	75.5		
N stage				455.331	<.001				505.318	<.001
N0	97.8	97.0	95.2			98.8	98.5	98.4		
N1	90.7	89.4	87.2			93.5	92.7	92.2		
N2	94.8	93.7	90.6			95.6	95.4	94.9		
N3	87.2	86.2	82.7			91.0	90.4	88.6		
M stage				1866.861	<.001				2192.811	<.001
M0	98.4	97.6	95.8			99.2	99.0	98.8		
M1	78.4	76.8	73.0			83.1	82.0	81.1		
SEER stage				1874.705	<.001				2171.302	<.001
Localized	98.8	98.1	96.3			99.5	99.3	99.2		
Regional	96.8	95.9	94.0			98.2	97.8	97.5		
Distant	78.8	77.0	73.3			83.4	82.3	81.4		
Surgery				166.335	<.001				155.316	<.001
No	62.1	60.8	58.1			74.2	74.2	74.2		
Yes	96.6	95.8	93.8			97.8	97.5	97.3		
Radiotherapy				26.456	<.001				19.854	<.001
Yes	97.5	96.8	95.4			98.4	98.3	98.1		
No	96.1	95.2	93.0			97.5	97.2	96.9		

Note: P-value<0.05 are shown in bold.

Abbreviations: AJCC, American Joint Committee on Cancer; SEER, Surveillance, Epidemiology, and End Results.

### Identification of prognostic factors of OS and CSS in GCTC patients

Univariate and multivariate Cox regression were used to analyze the related factors of OS and CSS in patients with GCTC. In the training cohort, univariate Cox regression analysis showed that age at diagnosis, race, AJCC stage, T stage, N stage, M stage, SEER stage, surgery and radiotherapy were related factors of OS and CSS in GCTC patients. After all the above factors were included in the multivariate Cox regression analysis, we found that T stage, N stage, M stage and radiotherapy were not independent risk factors for OS, while N stage and surgery were not independent risk factors for CSS (Table 3).

**Table 3:** Univariate and multivariate analysis of overall survival (OS) and cancer-specific survival (CSS) rates in training cohort.

**Characteristic**	**OS**					**CSS**			
	**Univariate analysis**		**Multivariate analysis^a^**			**Univariate analysis**		**Multivariate analysis^b^**	
	**Hazard Ratio (95% CI)**	**P value**	**Hazard Ratio (95% CI)**	**P value**		**Hazard Ratio (95% CI)**	**P value**	**Hazard Ratio (95% CI)**	**P value**
Age at diagnosis									
0-20	Reference		Reference			Reference		Reference	
21-40	0.94 (0.70-1.27)	.677	1.25 (0.92-1.69)	.151		0.80 (0.55-1.17)	.246	1.19 (0.82-1.75)	.361
41-60	1.52 (1.11-2.08)	.008	2.27 (1.66-3.11)	<.001		1.11 (0.74-1.65)	.627	1.89 (1.26-2.83)	.002
> 60	5.11 (3.48-7.49)	<.001	7.60 (5.17-11.16)	<.001		2.34 (1.31-4.18)	.004	4.07 (2.27-7.29)	<.001
Race									
White	Reference		Reference			Reference		Reference	
Black	1.64 (1.12-2.39)	.011	1.49 (1.02-2.18)	.040		1.26 (0.69-2.31)	.445	1.16 (0.64-2.12)	.626
Others	1.11 (0.83-1.48)	.481	1.26 (0.94-1.68)	.121		1.46 (1.02-2.08)	.036	1.71 (1.19-2.44)	.003
AJCC stage									
I	Reference		Reference			Reference		Reference	
II	1.50 (1.15-1.96)	.003	0.90 (0.60-1.35)	.606		2.38 (1.57-3.60)	<.001	0.96 (0.52-1.75)	.882
III	8.21 (7.03-9.58)	<.001	1.81 (1.14-2.89)	.012		20.54 (16.07-26.25)	<.001	2.22 (1.08-4.55)	.029
T stage									
T1	Reference		Reference			Reference		Reference	
T2	1.32 (1.12-1.57)	.001	-	.918		1.41 (1.10-1.79)	.006	0.97 (0.76-1.24)	.811
T3	3.46 (2.75-4.34)	<.001	-	.365		4.45 (3.29-6.00)	<.001	1.03 (0.75-1.40)	.879
T4	10.11 (6.75-15.16)	<.001	-	.016		16.23 (10.11-26.04)	<.001	2.33 (1.43-3.80)	.001
N stage									
N0	Reference		Reference			Reference		Reference	
N1	3.16 (2.62-3.80)	<.001	-	.407		5.06 (3.93-6.52)	<.001	-	.831
N2	2.00 (1.52-2.64)	<.001	-	.034		3.41 (2.40-4.83)	<.001	-	.204
N3	4.13 (3.26-5.22)	<.001	-	.721		7.45 (5.56-9.98)	<.001	-	.442
M stage									
M0	Reference		Reference			Reference		Reference	
M1	8.95 (7.71-10.38)	<.001	-	.109		19.13 (15.43-23.72)	<.001	2.14 (0.90-5.08)	.084
SEER stage									
Localized	Reference		Reference			Reference		Reference	
Regional	1.81 (1.46-2.24)	<.001	1.81 (1.28-2.55)	.001		3.40 (2.41-4.79)	<.001	3.10 (1.80-5.34)	<.001
Distant	10.38 (8.82-12.23)	<.001	6.19 (3.80-10.08)	<.001		28.26 (21.61-36.94)	<.001	6.86 (2.69-17.49)	<.001
Surgery									
No	Reference		Reference			Reference		Reference	
Yes	0.06 (0.03-0.12)	<.001	0.28 (0.13-0.61)	.001		0.05 (0.02-0.14)	<.001	-	.829
Radiotherapy									
Yes	Reference		Reference			Reference		Reference	
No	1.54 (1.27-1.87)	<.001	-	.930		1.53 (1.15-2.03)	.003	0.67 (0.50-0.91)	.010

Abbreviations: OS, Overall survival; CSS, Cancer-specific survival; AJCC, American Joint Committee on Cancer; SEER, Surveillance, Epidemiology, and End Results.

^a^Model was adjusted by age at diagnosis, race, AJCC stage, TNM stage, SEER stage, surgery and radiotherapy.

^b^Model was adjusted by age at diagnosis, race, AJCC stage, TNM stage, SEER stage, surgery and radiotherapy.

### Prognostic nomograms for OS and CSS

**Figure 1:**
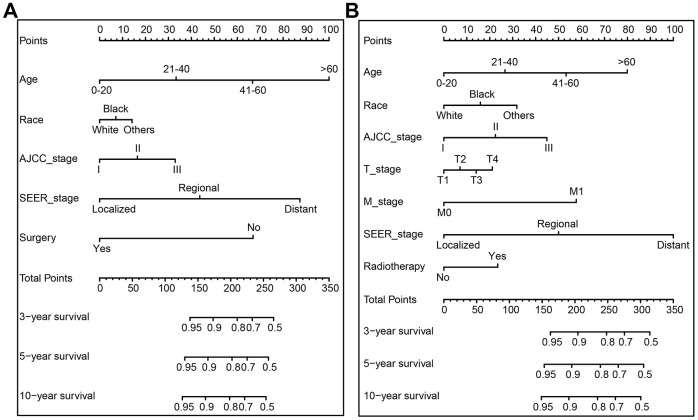
**The nomogram predicting 3-, 5-, and 10-year overall survival (OS) and cancer-specific survival (CSS) rate of GCTC patients the training cohort.** (**A**) OS nomogram; (**B**) CSS nomogram.

In the training cohort, we developed and established two nomograms for OS and CSS: one nomogram of independent risk factors associated with prognosis based on the multivariate Cox regression analysis, and the other one nomogram based on TNM stage. Figure 1 and Supplementary Figure 1 (TNM stage) show the nomogram of the prognosis of 3-, 5- and 10-year OS and CSS. Each subtype of the variables on the nomogram corresponds to a point on the "Point" scale. By adding the scores associated with each variable and projecting the "Total point" to the lowest number, the probabilities of OS and CSS for 3-, 5-, and 10- years can be estimated.

The length of the line corresponding to each variable in the nomogram represents the influence of the predictive variable on the survival outcome. We found that for nomogram generated by multivariate Cox regression analysis, age contributed the least to survival outcome in the OS nomogram, and SEER stage has the greatest contribution to the survival outcome in the nomogram of CSS, followed by age (Figure 1). Regardless of the OS or CSS nomogram generated by T stage, N stage and M stage, M stage made the greatest contribution to survival outcome.

### Validation and calibration of the nomograms

**Figure 2:**
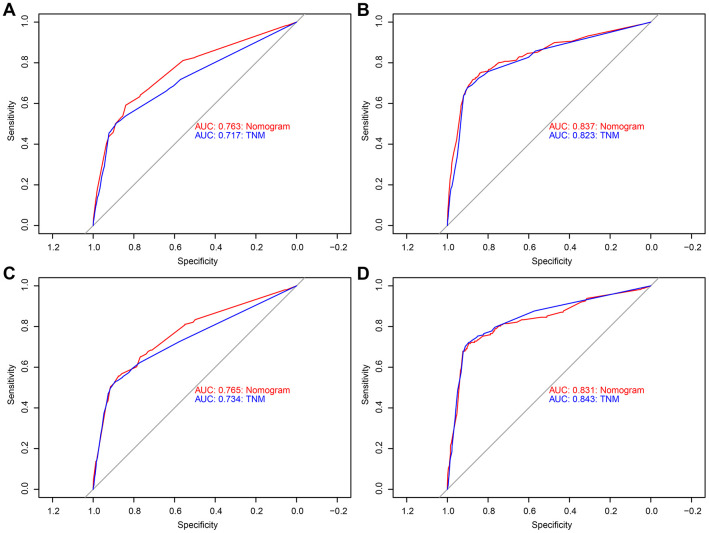
**Receiver operating characteristic (ROC) curves detects the predictive value of two nomograms in GCTC prognosis.** (**A**) Overall survival (OS) the training cohort. (**B**) Cancer-specific survival (CSS) the training cohort. (**C**) OS the validation cohort. (**D**) CSS the validation cohort.

**Figure 3:**
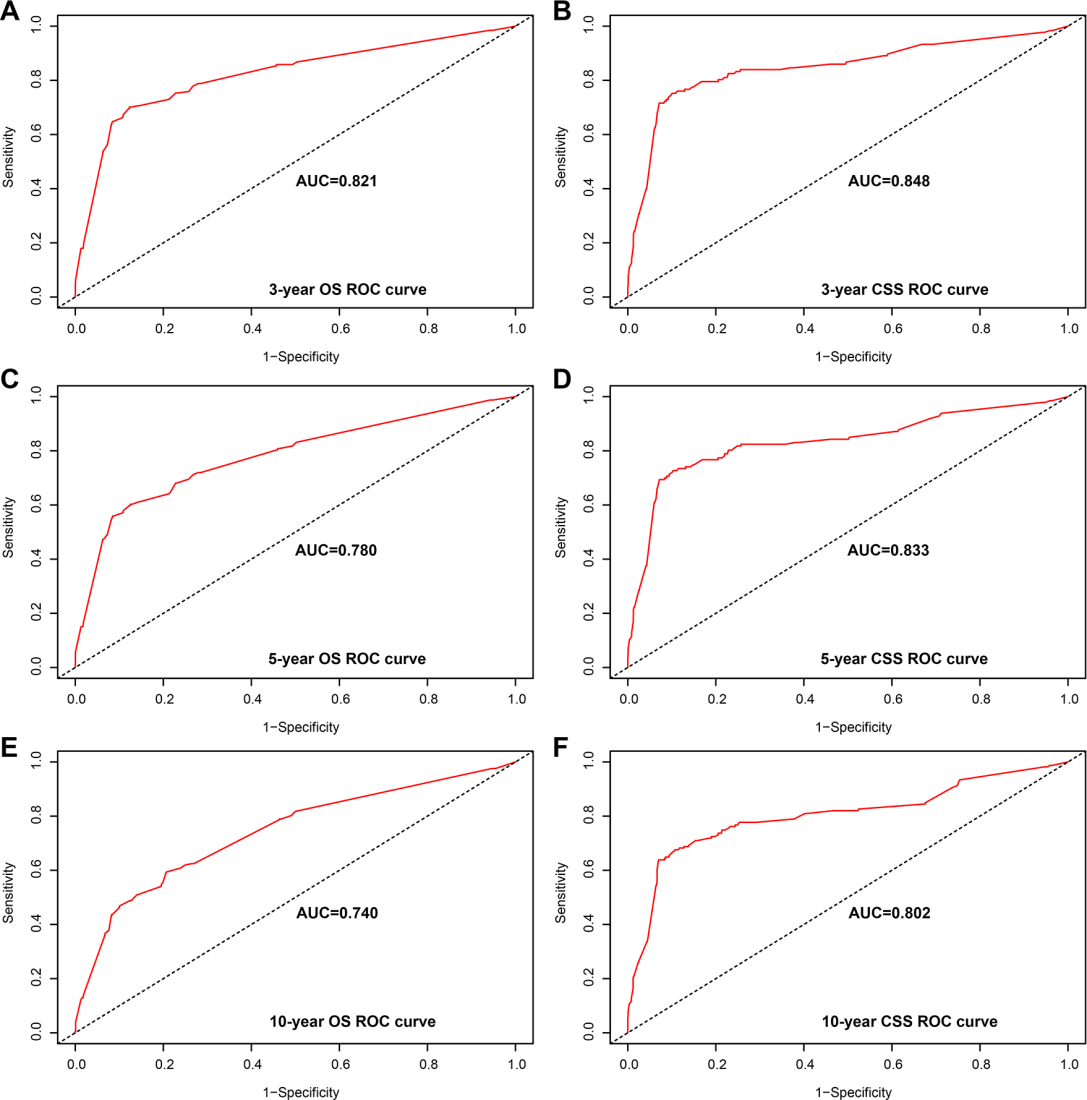
**Area under the curve (AUC) value of the receiver operating characteristic (ROC) predicting in the training cohort.** (**A**) 3-year overall survival (OS) rates. (**B**) 3-year cancer-specific survival (CSS) rates. (**C**) 5-year OS rates. (**D**) 5-year CSS rates. (**E**) 10-year OS rates. (**F**) 10-year CSS rates.

Analysis of the time-dependent ROC curves for OS shows that the AUC for the ROC curve of the nomogram (training cohort: AUC=0.763; validation cohort: AUC=0.765) was significantly larger than that of TNM stage (training cohort: AUC=0.717; validation cohort: AUC=0.734), but the ROC curve of CSS was similar (Figure 2). Moreover, we evaluated the predictive performance of the nomogram for 3-, 5- and 10-year OS and CSS in the training and validation cohorts and found that the nomograms provided a good assessment of OS and CSS at 3-, 5- and 10-year in GCTC patients (Figure 3 and Supplementary Figure 2).

**Figure 4:**
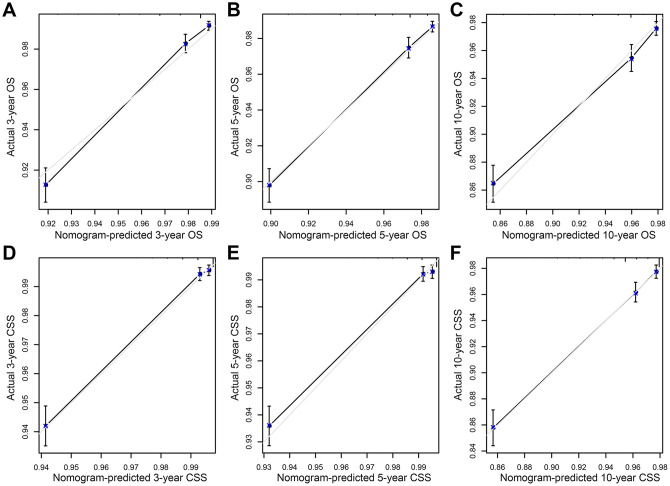
**Calibration plot of the nomogram for predicting 3-, 5-, and 10-year overall survival (OS) and cancer-specific survival (CSS) in training cohort.** (**A**) 3-year OS; (**B**) 5-year OS; (**C**) 10-year OS; (**D**) 3-year CSS; (**E**) 5-year CSS; (**F**) 10-year CSS.

In order to compare the predicted survival time with the actual survival time, the C-index and calibration curves were used to verify the nomogram in the training and validation cohorts. We found that the C-index of the nomogram OS was larger than that of the TNM stage nomogram (training cohort: nomogram=0.784, 95% CI: 0.765-0.803; TNM stage=0.746, 95% CI: 0.725-0.767, p<0.001; validation cohort: nomogram=0.785, 95% CI: 0.756-0.814; TNM stage=0.765, 95% CI: 0.735-0.795; p=0.003), while the C-index of CSS was basically same in both training and validation cohorts (training cohort: nomogram=0.844, 95% CI: 0.820-0.869; TNM stage=0.833, 95% CI: 0.809-0.857, p=0.053; validation cohort: nomogram=0.841, 95% CI: 0.804-0.878; TNM stage=0.854, 95% CI: 0.822-0.886; p=0.186). Moreover, we calibrated the 3-, 5- and 10-year OS and CSS nomogram of the training cohort and the validation cohort. The calibration curves in Figure 4 and Supplementary Figure 3 were very close to the perfect curves, showing a good consistency between the prediction of the nomogram and the actual observation in the training and validation cohort.

**Figure 5:**
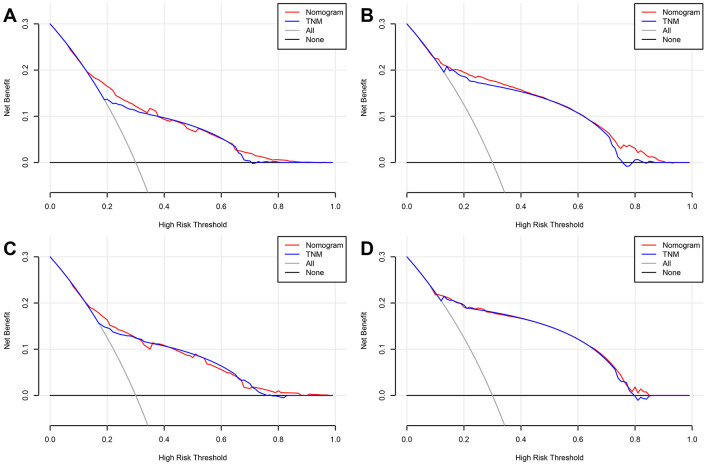
**Decision curve analysis (DCA) curves detects the predictive value of two nomograms in GCTC prognosis.** (**A**) Overall survival (OS) in the training cohort. (**B**) Cancer-specific survival (CSS) in the training cohort. (**C**) OS in the validation cohort. (**D**) CSS in the validation cohort.

**Figure 6:**
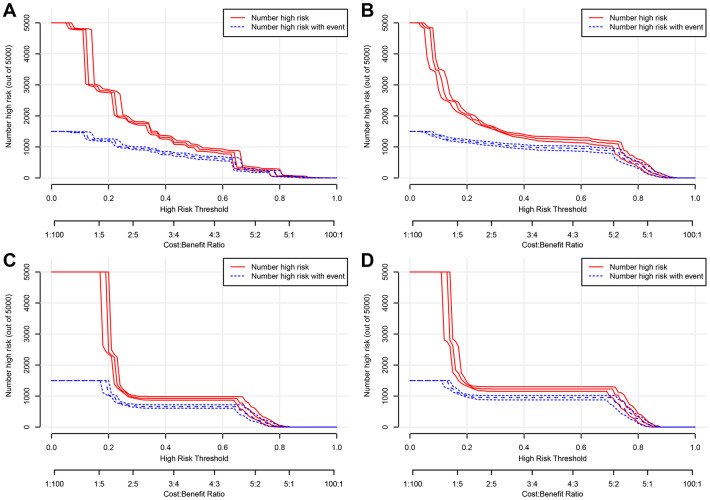
**Clinical impact curve (CIC) detects the predictive value of two nomograms in GCTC prognosis in the training cohort. (A**) The overall survival (OS) of the nomogram. (**B**) The OS of the TNM stage. (**C**) The cancer-specific survival (CSS) of the nomogram. (**D**) The CSS of the TNM stage.

In addition, DCA calculated the net benefit to evaluate the clinical utility of the nomograms. The results showed that in a wide range of OS thresholds the clinical net benefit of the nomograms generated by multivariate Cox regression analysis was greater than the nomograms produced by TNM stage (Figure 5) in both training and validation cohorts. Both in training cohort and validation cohort, the CIC results show that the multivariate Cox regression analysis produced nomograms that were classified as positive among the broad thresholds for OS, and the number of true positives was greater than the TNM stage nomograms (Figure 6 and Supplementary Figure 4).

## DISCUSSION

In this study, we first established prognostic nomograms of OS and CSS in patients with GCTC. We performed Cox regression analysis on a large number of GCTC patients using the SEER database and found that age at diagnosis, race, AJCC stage and SEER stage were independent risk factors for OS and CSS. We constructed two prognostic nomograms: one based on multivariate Cox regression analysis and the other one TNM stage. By examining the C-index, ROC curve, DCA curve and CIC we found that the nomogram based on multivariate Cox regression analysis has better OS prognosis than the TNM stage nomogram and has similar prognostic ability in CSS. In addition, we have verified and calibrated the established nomograms and have evaluated the accuracy of the OS and CSS alignment charts for 3-, 5- and 10-year. The results show that there was a good consistency between the prediction of the nomograms and the actual observation and also good reliability in both internal and external verification.

TNM staging classification system was the most versatile tumor staging system in the world and also the foundation of GCTC prognosis [11]. The TNM stage was determined based on the results of laboratory tests and postoperative pathological examinations [12]. In this classification system, clinicians determine TNM stage based on the depth of tumor invasion (T), number of lymph node metastasis (N) and distant metastasis (M). For cancer patients with different TNM stage high stage means complex drug treatment and short survival time. How to better combine the patients’ tumor characteristics and their own clinical factors to make a tailored assessment of the risk of patients has been a challenge for clinicians [13].

The nomogram was a predictive tool, which was a graphical representation based on multivariate prognostic regression analysis, making the prognostic factors more visual [14, 15]. The model integrates variety of prognostic factors and is well prepared to evaluate the survival probability of individual patients [16]. At present, many cancer nomograms have been developed and shown more accurate predictions of the cancer prognosis than traditional TNM systems [17, 18]. In addition, nomograms allow clinicians to incorporate more prognostic factors and assess the patient's physical condition more intuitively in order to evaluate the personalized prediction for clinical trial participation. Therefore, it was of great significance to establish an effective and reliable nomogram for the prognosis of patients with GCTC and to provide them with individualized treatment.

The nomogram has been widely used in various urinary malignancies, which was of great significance for individualized and accurate prediction of prognosis [19–21]. Karakiewicz et al. [22] performed preoperative prediction of 726 patients treated with radical cystectomy and bilateral pelvic lymphadenectomy, and found that the multivariate nomogram was more accurate than the TUR T stage alone prediction. Similarly, kattan et al. [23] constructed a nomogram that included pre-treatment serum prostate-specific antigen levels, biopsy Gleason scores and clinical stages, and found that it could predict the 5-year treatment failure probability with clinically localized prostate cancer who underwent radical prostatectomy. Zhou et al. [24] found that nomogram and Aggtrmmns scoring system can effectively predict kidney cancer patient's OS and CSS. In our study, we developed a nomogram based on age, race, AJCC stage, TNM stage, SEER stage, surgery and radiotherapy variables, and the nomogram showed better ability to predict the prognosis than the TNM stage nomogram. Using this nomogram, urologists can evaluate the prognostic survival of patients with GCTC, enabling personalized treatment and monitoring possible. There are limitations to be recognized in this study. First, this study was a retrospective study with limitations, sample and ethnic selection bias and more cases needed for prospective studies. Second, the SEER database has certain limitations regarding type/duration of treatment and recurrence of disease and we cannot obtain detailed specific information (dose, beam energy and fractionation) of radiotherapy. Moreover, the information on the patient's physical condition and complications is lacking, both of which are prognostic factors for patients with GCTC.

Based on a large number of population data, we developed prognostic nomogram for GCTC patients, which can accurately and reliably predict the 3-, 5- and 10-year OS and CSS in individual GCTC patients. The proposed GCTC survival model can help clinicians make personalize therapies and adjust follow-up strategies.

## MATERIALS AND METHODS

### Patients selection

The data presented in our study were retrieved from the Surveillance Epidemiology and End Results (SEER) database, which funded by the National Cancer Institute. The SEER database covers approximately 28% of the US population and includes demographic information and cancer characteristics, such as diagnosis age, insurance status, race, marital status, income status, primary tumor site, tumor grade and stage, histological type, Tumor-Node-Metastasis (TNM) stage, treatment modality and survival time [25]. The National Cancer Institute's SEER*Stat software version 8.3.5 (https://seer.cancer.gov/seerstat/)(SEER 18 Regs Custom Data (with additional treatment fields), Nov 2018 Sub (1975-2016 varying) database) was used in this study.

The *International Classification of Diseases for Oncology* (ICD-O) site codes C62.1 and C62.9 were used to identify patients diagnosed with TC between 2004 and 2015. We collected 26,780 GCTC patients according to the ninth edition of the International Classification of Diseases (codes: 9061 to 9064, 9070 to 9071, 9080 to 9085 and 9100 to 9102). Exclusion criteria in our study were as follows: (a) unknown T stage and T0 (n=1,808); (b) unknown N stage (n=831); (c) unknown M stage (n=118); (d) not one primary tumor only (n=1,948). All patients were followed up until December 2016 to ensure that all cases were observed for more than one year at the last follow-up. Finally, we left 22,165 eligible patients diagnosed with GCTC (Supplementary GCTC data).

### Study variables

Variable definition information about age at diagnosis, race, AJCC stage, T stage, N stage, M stage, SEER stage, surgery, radiotherapy, cause of death and survival time can be found in the SEER database. The starting point of the follow-up was the date of diagnosis of GCTC, and the end point was cancer-specific death or the last follow-up in December 2016. The overall survival (OS) time corresponded to the length of time from the date of diagnosis to the death from any cause or the date on which data were censored. During analyzing cancer-specific survival (CSS), mortality cases associated with other causes were excluded.

### Statistical analysis

We randomly assigned 70% of patients to the training cohort (n=15,515) and the remaining 30% to the validation cohort (n=6,650). Kaplan-Meier curve was used to estimate the OS and CSS of GCTC, and the difference between the curves was analyzed by log-rank test. Univariate and multivariate Cox regression models were performed to estimate the hazard ratios (HR) and 95% confidence intervals (CI) to analyze independent prognostic factors of GCTC.

Using R software, we constructed two nomograms: one nomogram the multivariate Cox regression analysis, and the other one nomogram based on TNM stage, to predict the OS and CSS probabilities of individual patients. We first used the R software to generate the receiver operating characteristic (ROC) curve for the two nomograms and determined the area under the curve (AUC). In addition, by comparing the predicted survival time with the observed survival time, the predictive performance of the nomogram was evaluated using the consistency index (C-index) and calibration curve, and the nomogram was calibrated for 3-, 5- and 10-years OS and CSS. The C index was similar to the AUC, but seems to be more suitable for censored data. The value of the C-index statistic was between 0.5 (non-discrimination) and 1 (perfect discrimination), and a higher C-index value indicates a better prognostic model. These evaluations were performed using a bootstrap with 1000 resamples.

There was no direct clinical interpretation for C-index. Therefore, we also analyzed the decision curve analysis (DCA), which is a novel method to evaluate the predictive model for evaluating net benefits from the perspective of clinical outcome, and plotted the clinical impact curve (CIC) based on the results of DCA.

The above statistical analysis uses SPSS version 20.0 (SPSS, Chicago, USA) and statistical software package R version 3.5.3 (http://www.r-project.org/) and R software packages rms, survival, formula, ggplot2 and rmda. P-value ≤ 0.05 (two-sided) was considered statistically significant.

## Supplementary Material

Supplementary Figures

Supplementary Data
