# Supplementary material for: Development and validation of prognostic nomogram for germ cell testicular cancer patients
Source: Aging (Albany NY). 2020 Nov 2;12(21):22095–111. doi: 10.18632/aging.104063 (PMC7695357; doi:10.18632/aging.104063)
Supplement: Supplementary Figures [file aging-12-104063-s001..pdf]

Supplementary Figures

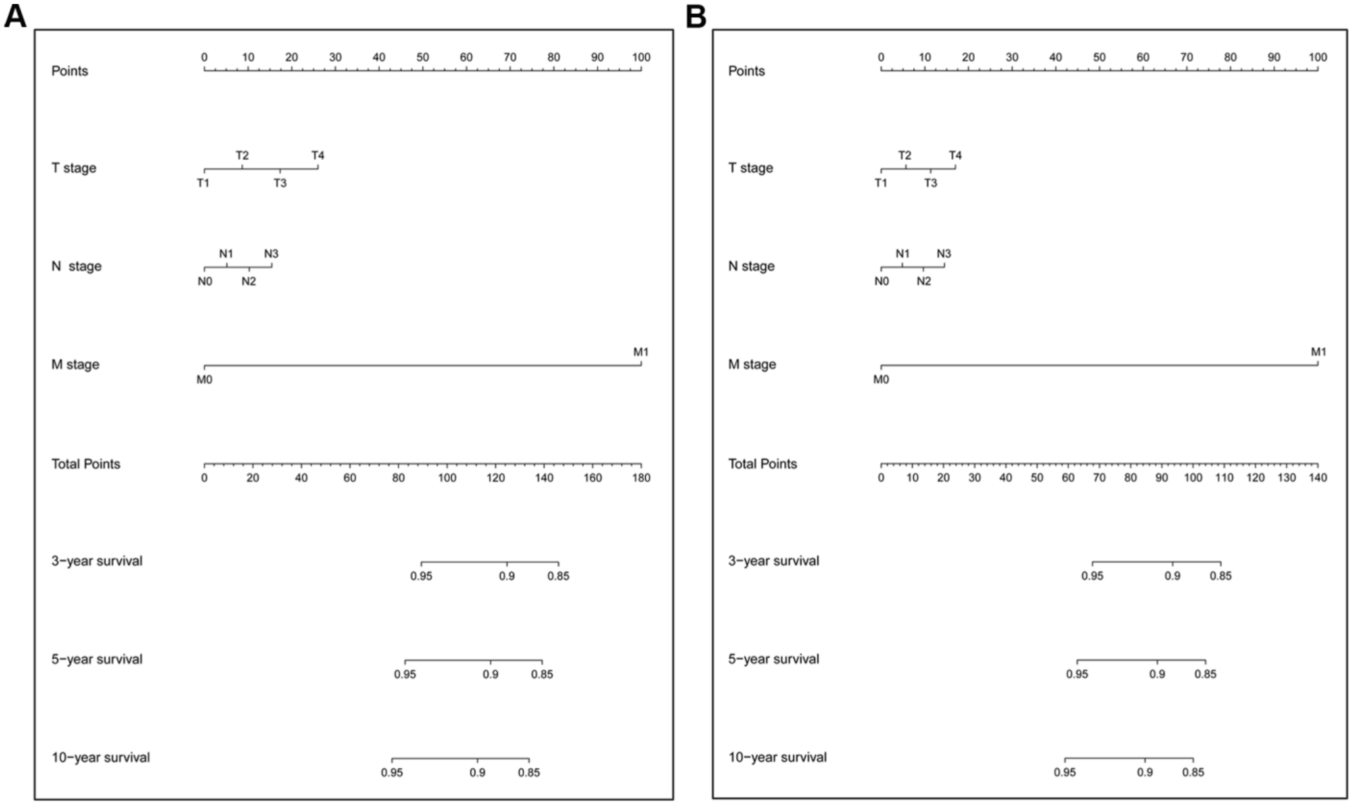

Supplementary Figure 1. TNM stage nomogram predicting 3-, 5-, and 10-year overall survival (OS) and cancer-specific survival (CSS) rate of GCTC patients. (**A**) OS nomogram; (**B**) CSS nomogram.

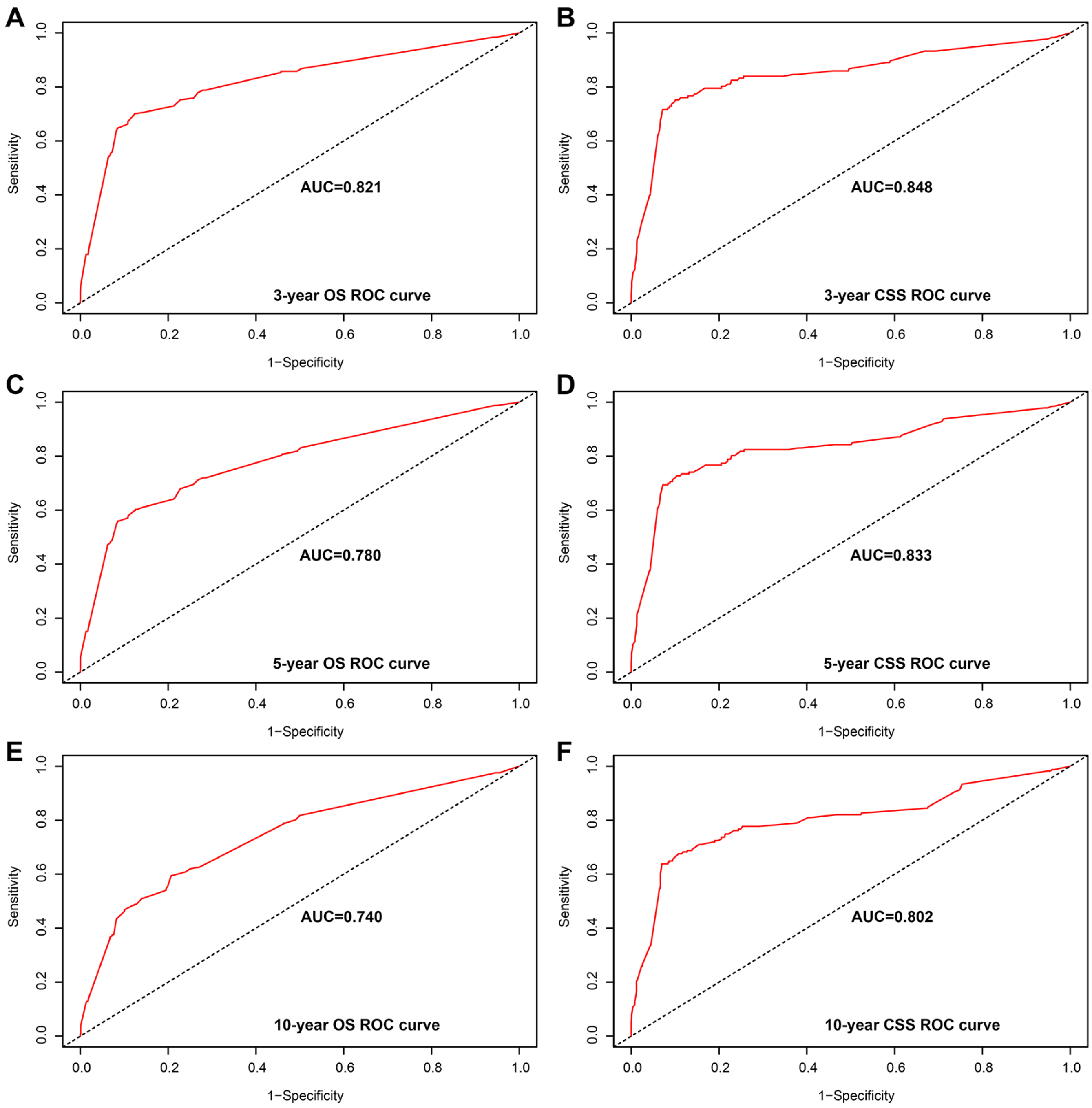

Supplementary Figure 2. Area under the curve (AUC) value of the receiver operating characteristic (ROC) predicting in the validation cohort. (**A**) 3-year overall survival (OS) rates. (**B**) 3-year cancer-specific survival (CSS) rates. (**C**) 5-year OS rates. (**D**) 5-year CSS rates. (**E**) 10-year OS rates. (**F**) 10-year CSS rates.

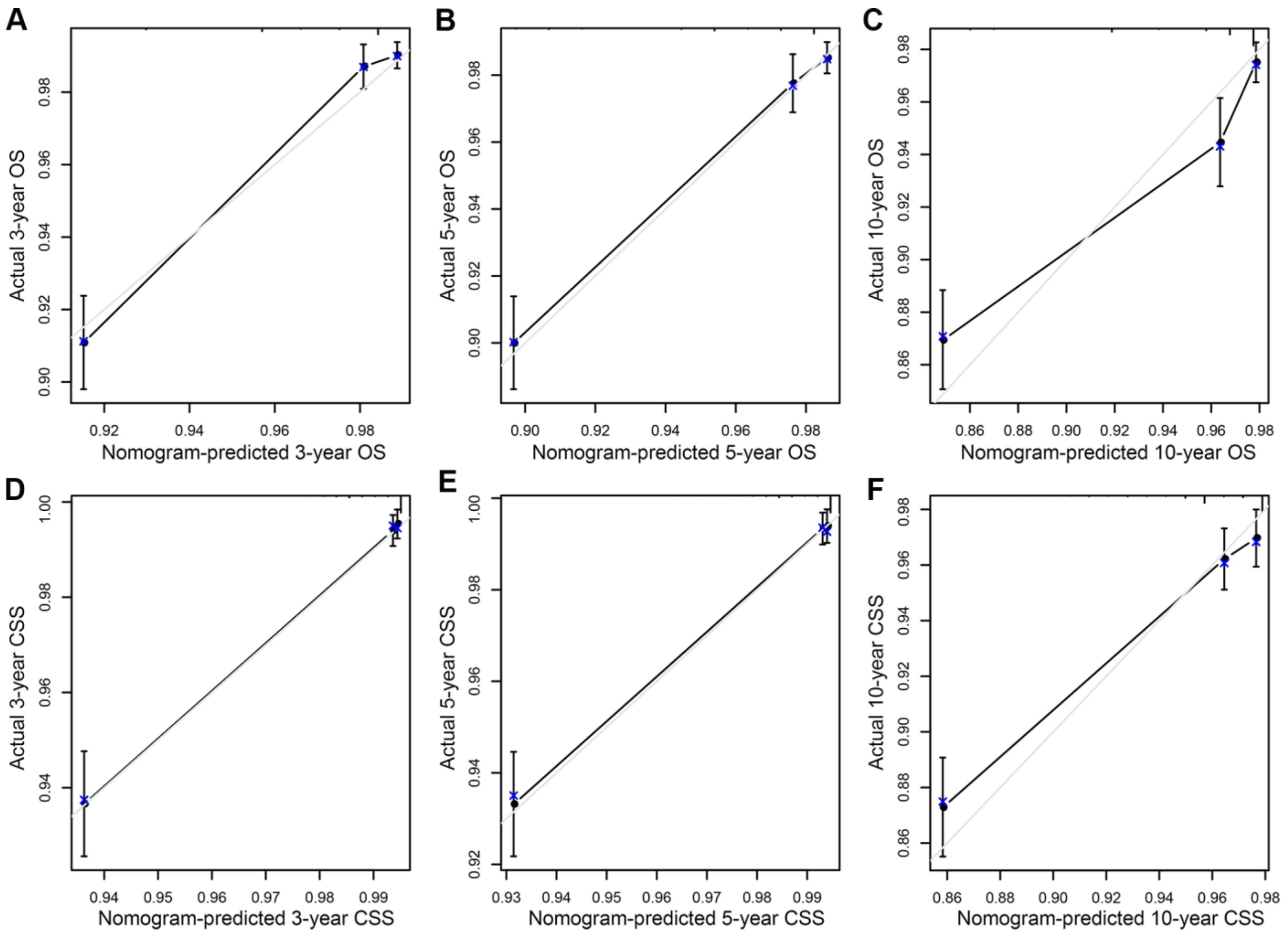

Supplementary Figure 3. Calibration plot of the nomogram for predicting 3-, 5-, and 10-year overall survival (OS) and cancer-specific survival (CSS) in validation cohort. (**A**) 3-year OS; (**B**) 5-year OS; (**C**) 10-year OS; (**D**) 3-year CSS; (**E**) 5-year CSS; (**F**) 10-year CSS.

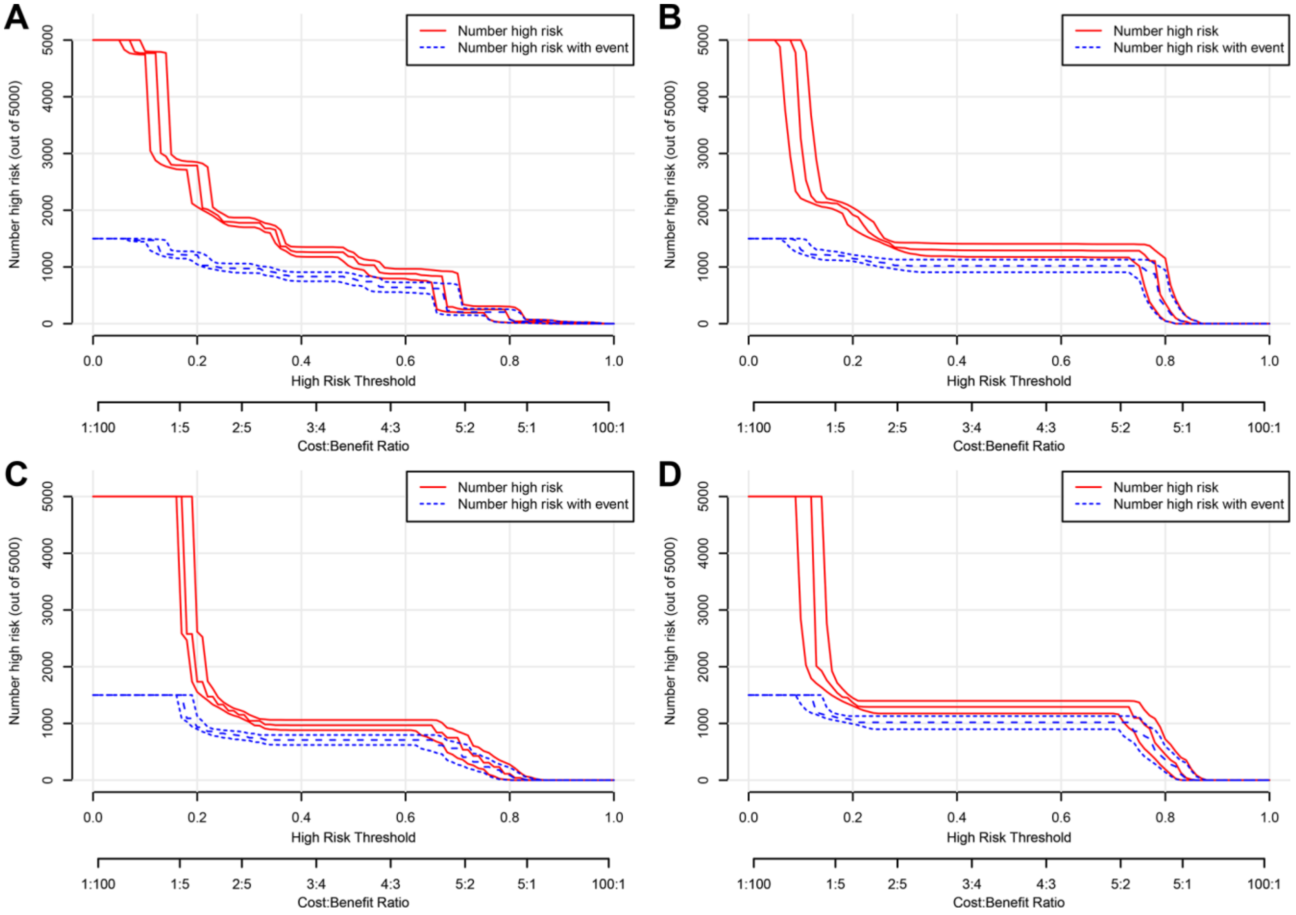

Supplementary Figure 4. Clinical impact curve (CIC) detects the predictive value of two nomograms in GCTC prognosis in the validation cohort. (**A**) The overall survival (OS) of the nomogram. (**B**) The OS of the TNM stage. (**C**) The cancer-specific survival (CSS) of the nomogram. (**D**) The CSS of the TNM stage.
